# Small Bowel Volvulus: A Case Report

**DOI:** 10.7759/cureus.1281

**Published:** 2017-05-28

**Authors:** Tushar Patial, Sahil Chaddha, Namit Rathore, Vishal Thakur

**Affiliations:** 1 General Surgery, Indira Gandhi Medical College, Shimla; 2 Medical Student, Drexel University College of Medicine

**Keywords:** abdominal pain, case report, intestinal volvulus, small bowel volvulus, midgut volvulus, small bowel obstruction

## Abstract

Small bowel volvulus is a rare clinical entity which presents as recurrent intermittent abdominal pain after consumption of food. Although the entity is well described in the literature, diagnosis is often difficult due to its clinical presentation being similar to mesenteric ischemia. Herein we present the case of a 44-year-old male who presented to us with this condition.

## Introduction

Small bowel volvulus (SBV) refers to the abnormal twisting of a loop of small bowel around the axis of its own mesentery [1]. Recurrent, intermittent periumbilical or epigastric pain occurring after ingestion of a meal with severity out of proportion to clinical examination is an important clinical finding [2-3]. Because clinical presentation is similar to mesenteric ischemia, it is important to differentiate the two entities, since their management is different. Informed consent statement was obtained for this study.

## Case presentation

**Figure 1:**
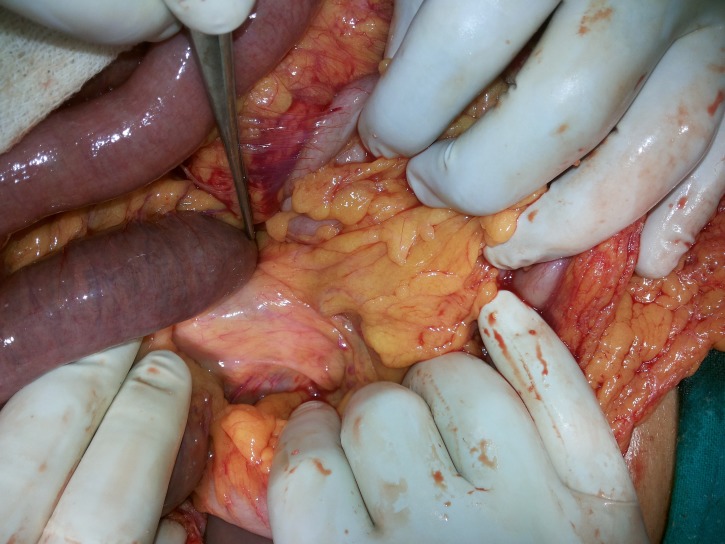
“Clockwise” twist of the ileum with instrument pointing at point of rotation

A 44-year-old male presented to the emergency with the chief complaints of recurrent postprandial epigastric pain for three days. On scrutiny of previous records, it was discovered that this was his third hospitalization for the same complaint. On all three occasions, the patient reported that his complaints were triggered after fasting for religious reasons. He had also undergone a gastroduodenoscopy three months back revealing gastric erosions for which he had been prescribed omeprazole. At the time of the admission, this was the only drug he had been taking. No history of previous abdominal surgery was noted. On examination, apart from tachycardia, other vitals were stable. The abdomen was distended but tenderness could not be elicited. On digital rectal examination, there was rectal ballooning without any soiling of the examining finger. All hematological investigations were within normal limits and an ultrasound of the abdomen was also normal. The abdominal radiograph showed multiple air fluid levels suggestive of intestinal obstruction. A computed tomography of the abdomen was also suggestive of subacute intestinal obstruction. The patient was started on intravenous fluids and a nasogastric tube was inserted. Twelve hours later, the patient underwent an exploratory laparotomy for worsening abdominal pain, signs of peritonitis and non-resolving small bowel obstruction. On opening the abdomen, the ileum had twisted along its mesenteric axis in the clockwise direction (Figure 1).

**Figure 2:**
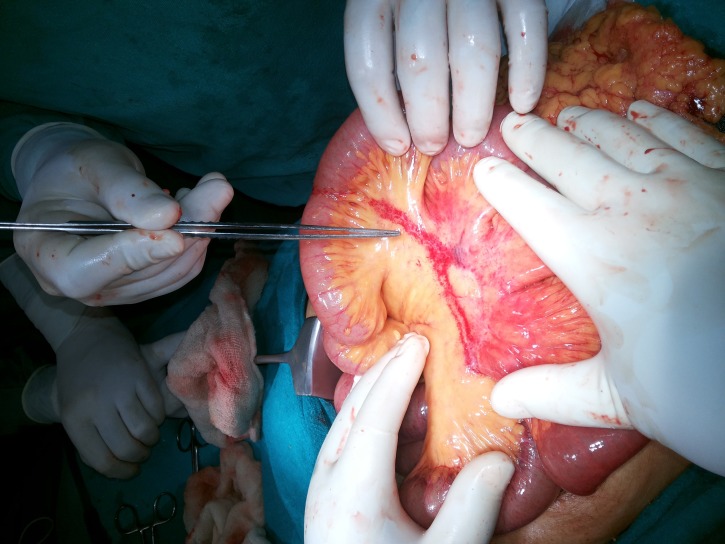
Untwisted and viable bowel with line of rotation along mesentery

Mild distention of the stomach and proximal ileal loops was noted. The large bowel distal to the site of twisting was collapsed. No perforation or band was found. The volvulus was untwisted, and a line of rotation was seen. No fixation of the twisted segemnt was done. The bowel was slightly discoloured, but viable and started to return to its normal colour and shine (Figure 2).  

The patient was given 100% oxygen and the bowel was covered with warm moist sponges till the serosa returned to its normal color. Further examination revealed no other abnormalities like internal hernias or malrotation. The post-operative period was uneventful and the patient was discharged on the fourth postoperative day.

## Discussion

In our patient, the diagnosis of small bowel volvulus was made in the operating room. Recurrent episode of pain and non-specific findings on computed tomography made the diagnosis even more difficult. Small bowel volvulus (SBV) is rare in western countries (annual incidence; 1.7–5.7 per 100,000 population) but has the higher incidence in the Middle East, Asia and Africa (annual incidence; 24–60 per 100,000 population) [4]. Risk factors include low socioeconomic status, diabetic autonomic neuropathy, and parasitic infestation. It has also been reported that sudden changes in dietary habits with ingestion of food after long fasting is the known risk factor. This was the precipitating factor in our patient. It is believed that sudden overloading of an empty bowel by a single voluminous meal may induce forceful bowel peristalsis, resulting in small bowel volvulus [1]. Anatomical predisposition for the condition has also been proposed with patients with volvulus having longer mesenteries and shorter mesenteric attachments when compared with normal individuals [5- 6]. The entity may be primary or may occur secondarily to intestinal malrotation, adhesions, internal hernias, tumors, mesenteric lymph nodes, Meckel’s diverticulum, lipomas, pregnancy, endometriosis, tuberculosis, aneurysms and hematomas [7]. Clinically, sudden onset of signs and symptoms of small-bowel obstruction in a patient without previous abdominal surgery, with epigastric or periumbilical pain several days before, is suggestive of the diagnosis. Often, pain out of proportion to physical findings may be a presenting feature, further complicating its differentiation from acute mesenteric ischemia [7]. Since abdominal radiographs are often nonspecific, computed tomography is the choice of investigation. On computed tomography, signs suggestive of SBV include rotation of the mesentery and the mesenteric vessels, dilated gut loops and signs of intestinal ischemia such as the presence of air in the bowel wall, portal vein gas, and free peritoneal fluid [4, 8]. Complications include bowel obstruction, ischemia, and necrosis [1-2]. Although this diagnosis is rare, early surgical intervention is key, since gangrenous bowel is associated with high mortality (20%-100%) [2].

## Conclusions

A high index of suspicion is required to identify small bowel volvulus. Owing to its variable presentation and high mortality, all clinicians must consider SBV as a possible diagnosis in a patient with abdominal pain. Emergent computed tomography of the abdomen and early surgical intervention are key to avoid adverse outcomes.
